# Fate of Tricuspid Valve Five Years after Left Heart Valve Replacement

**DOI:** 10.1186/1749-8090-10-S1-A21

**Published:** 2015-12-16

**Authors:** Anbarasu Mohanraj, Saikumari Nagaraj, Rekha Arunagiri, Rajan Sethurathinam, VM Kurian

**Affiliations:** 1 Institute of Cardio Vascular Diseases, Madras Medical Mission, Chennai, India

## Background/Introduction

Residual functional tricuspid regurgitation can cause significant symptoms after a successful left heart valve replacement surgery. There is more evidence towards repairing moderate tricuspid valve regurgitation of late.

## Aims/Objectives

We analysed the fate of Tricuspid valve at the end of five years after a left heart valve replacement irrespective of whether they had undergone a concomitant TV repair or not during the initial surgery

## Method

Between January 2008 and December 2009, 200 patients who had undergone a left heart valve replacement were analysed for the degree of TV regurgitation at the end of five years. 162 patients had undergone a Mitral valve surgery and 38 patients had undergone a double valve replacement. Group I - 40 patients had a concomitant TV repair (Modified de Vaga's annuloplasty) during the primary surgery and Group II 160 patients did not have a concomitant TV repair.

## Results

In Group I, of the 40 patients, 4 patients (10%) underwent TV repair for moderate TV regurgitation, and all the 4 patients had trivial to mid TV regurgitation. 36 patients underwent TV repair for severe regurgitation, of these patients, 30 patients (83.3%) had trivial to mild residual TV regurgitation, while only 6 patients (16.6%) had moderate to severe TV regurgitation. In Group II, of the 160 patients, 58 patients (36.2%) and 16 patients (10%) had moderate and severe TV regurgitation which was left un-corrected. Of the 58 patients who had moderate TV regurgitation, 20 patients (34.4%) had moderate regurgitation and 4 patients (6.8%) had severe TV regurgitation at the end of five years. Of the 16 patients who had severe TR addressed during the initial surgery, 8 patients (50%) had moderate to severe TV regurgitation at the end of five years.

## Discussion/Conclusion

A concomitant Tricuspid valve repair is recommended during a left heart valve replacement, if the degree of Tricuspid regurgitation is moderate. This helps in alleviating the symptoms of residual tricuspid valve regurgitation on long term follow up.

